# Molecular species delimitation of shrub frogs of the genus *Pseudophilautus* (Anura, Rhacophoridae)

**DOI:** 10.1371/journal.pone.0258594

**Published:** 2021-10-19

**Authors:** Gajaba Ellepola, Jayampathi Herath, Kelum Manamendra-Arachchi, Nayana Wijayathilaka, Gayani Senevirathne, Rohan Pethiyagoda, Madhava Meegaskumbura

**Affiliations:** 1 College of Forestry, Guangxi Key Lab for Forest Ecology and Conservation, Guangxi University, Nanning, PR China; 2 Faculty of Science, University of Peradeniya, Peradeniya, Sri Lanka; 3 Postgraduate Institute of Archaeology, Colombo, Sri Lanka; 4 Department of Zoology, Faculty of Applied Sciences, University of Sri Jayewardenepura, Nugegoda, Sri Lanka; 5 Department of Organismal Biology and Anatomy, University of Chicago, Chicago, Illinois, United States of America; 6 Ichthyology Section, Australian Museum, Sydney, New South Wales, Australia; Sichuan University, CHINA

## Abstract

Sri Lanka is an amphibian hotspot of global significance. Its anuran fauna is dominated by the shrub frogs of the genus *Pseudophilautus*. Except for one small clade of four species in Peninsular India, these cool-wet adapted frogs, numbering some 59 extant species, are distributed mainly across the montane and lowland rain forests of the island. With species described primarily by morphological means, the diversification has never yet been subjected to a molecular species delimitation analysis, a procedure now routinely applied in taxonomy. Here we test the species boundaries of *Pseudophilautus* in the context of the phylogenetic species concept (PSC). We use all the putative species for which credible molecular data are available (nDNA–Rag-1; mt-DNA– 12S rRNA, 16S rRNA) to build a well resolved phylogeny, which is subjected to species delimitation analyses. The ABGD, bPTP, mPTP and bGMYC species delimitation methods applied to the 16S rRNA frog barcoding gene (for all species), 12S rRNA and Rag-1 nDNA grouped *P*. *procax* and *P*. *abundus*; *P*. *hallidayi* and *P*. *fergusonianu*s; *P*. *reticulatus* and *P*. *pappilosus*; *P*. *pleurotaenia* and *P*. *hoipolloi*; *P*. *hoffmani* and *P*. *asankai*; *P*. *silvaticus* and *P*. *limbus*; *P*. *dilmah* and *P*. *hankeni*; *P*. *fulvus* and *P*. *silus*.. Surprisingly, all analyses recovered 14 unidentified potential new species as well. The geophylogeny affirms a distribution across the island’s aseasonal ‘wet zone’ and its three principal hill ranges, suggestive of allopatric speciation playing a dominant role, especially between mountain masses. Among the species that are merged by the delimitation analyses, a pattern leading towards a model of parapatric speciation emerges–ongoing speciation in the presence of gene flow. This delimitation analysis reinforces the species hypotheses, paving the way to a reasonable understanding of Sri Lankan *Pseudophilautus*, enabling both deeper analyses and conservation efforts of this remarkable diversification. http://zoobank.org/urn:lsid:zoobank.org:pub:DA869B6B-870A-4ED3-BF5D-5AA3F69DDD27.

## Introduction

As a part of the Sri Lanka-Western Ghats biodiversity hotspot, Sri Lanka features an exceptional species richness and endemism in amphibians [1, 2]. At present, about 120 amphibian species are recognized from the island, of which 104 are endemic. Nineteen species are considered extinct, while 72 are threatened with extinction due to a plethora of human induced activities [3].

The family Rhacophoridae, spread across tropical to sub-tropical Asia and Africa, is a diverse group of frogs containing some 432 species, 6% of the world’s anuran fauna [4, 5]. Their taxonomy, evolution and biogeography have been studied in several recent analyses [2, 6–12]; much of this work has involved molecular analyses. Despite this, molecular data are available for only about 73% of the species (Genbank, last accessed January 2021). This highlights the fact that rhacophorid taxonomy and systematics could still benefit from species-level analyses [10]. Accuracy of species identification, together with a clearly resolved taxonomy, are critical to biological research, especially in evolution, ecology, conservation, and biogeography [13].

Rhacophorid tree frogs of the genus *Pseudophilautus*, characterized by terrestrial direct development [7], represent about 63% of amphibian species recognized from Sri Lanka. Such high diversity has been generated over the course of their complex evolutionary history, spanning ~30 MY [9, 11]. The key innovation of terrestrial direct development, together with factors such as climatic fluctuations, topography, orogeny, ecological opportunity and terrestrial connectivity with India mediated by sea-level fluctuations, have shaped this remarkable diversification [2, 7].

The genus *Pseudophilautus* currently contains 59 extant species in Sri Lanka [5]. Many of these were described relatively recently [14–21]. Some of these species are cryptic and hence difficult to identify from morphology alone, though validated in molecular analyses; others (e.g., [20, 21]) are based on morphological data alone. As of now, molecular data are available, at least for a single locus, for 49 of the 59 extant Sri Lankan *Pseudophilautus* (See S1 Table). However, population level data remains scarce for many of these species.

Though some species have been validated using molecular data, sometimes through an integrative approach, a detailed multi-gene phylogeny became available for them only recently [2]. Meanwhile, species delimitation analyses carried out recently [10] on a part of the previously available data, mostly based on singletons of the popular 16S frog barcoding gene [22, 23], suggested that a few closely related species of *Pseudophilautus*, e.g., *P*. *hankeni* with *P*. *dilmah and P*. *schmarda; P*. *papillosus* with *P*. *reticulatus* (and several species from other genera) do not reach the molecular thresholds of species delimitation in Rhacophoridae [10].

The initial descriptions of *Pseudophilautus* were often based on a few samples drawn from a few populations, which may not have captured the full breadth of the molecular and morphological variation of the species. With most species having been diagnosed from morphology alone, an objective assessment of species boundaries is needed to stabilize the species-level taxonomy of the group.

*Pseudophilautus* has been shown to evolve at a moderate and constant rate [2], a pattern now shown to hold true for all rhacophorids [10]. The slower and predominantly allopatric mode of speciation, the narrow-range endemism characteristic of many species, and the relatively small genetic distances that separate morphologically distinct species, seem to be characteristic of Rhacophoridae [2]. These factors too, need to be considered in evaluating the species boundaries of this diversification.

Molecular species delimitation analyses are now being increasingly used to inform taxonomic judgements [10, 24–29]. The goal of these analyses is to build a taxonomic scheme for a set of reliably-identified samples and infer a de novo delimitation of operational taxonomic units (OTUs) [27, 29, 30–33] in the context of the phylogenetic species concept (PSC). Currently, many molecular species delimitation methods exist, which are based on either molecular-distance or gene-tree approaches. Widely used methods include automatic barcode gap discovery (ABGD; [34]), the generalized mixed Yule-coalescent model (GMYC; [35, 36]), the Poisson tree processes model (PTP; [37, 38]), the Bayes factor delimitation [25, 39], the Bayesian coalescent method in the software Bayesian Phylogenetics and Phylogeography (BPP; [26]), and phylogeographic inference using approximate likelihoods [27]. However, these algorithm-based species delimitation approaches have their own limitations and been subjected to criticism [40]. Despite the uncertainty surrounding their results, these methods are still being used in delimiting species. They are used either as stand-alone methods or as integrative contributors to objective species delimitation [28, 29, 41].

Here we address the following questions in the context of *Pseudophilautus*. How many species meet the validity of molecular delimitation methods? Are there commonalities in distribution patterns of the sister taxa that fail delimitation thresholds? Which clades harbor species difficult to identify from morphology alone? What lesson can a study such as this contribute to traditional morphology-based taxonomy?

Here, using all available validated molecular data for *Pseudophilautus*, we test the validity of all species for which molecular data are available, using multiple molecular delimitation methods. We show that several nominal species fail to reach delimitation thresholds. We also show the existence of several potential new species, underscoring that *Pseudophilautus* would benefit from further taxonomic assessment.

## Materials and methods

### Ethics statement

Ethical review and approval is not applicable for this study as only published sequences from previous taxonomic and evolutionary studies on *Pseudophilautus* are used here.

### Obtaining molecular sequence data

We included the 104 individualsof *Pseudophilautus* previously analysed by [14–20], for which molecular data were available in Genbank, and also provided in [2]. Initially, we retrieved all the molecular data under the key words “Pseudophilautus” and “Philautus” in Genbank, along with their specific voucher numbers, and pooled these with the data provided by [2]. We followed the nomenclature of [5] for *Pseudophilautus* and curated the initial data set by removing duplicated sequences from the same specimen and misidentifications. The vouchered specimens of the species described since 2005 are based on topotypes from the original descriptions or from Meegaskumbura et al. 2019 [2]. The final data set contained 104, 83 and 60 sequences for the 16S, 12S mitochondrial loci and the nuclear Rag-1 locus, respectively. We ensured that the nominal species included in our data set could be identified through the voucher numbers provided in their original descriptions in order to match our molecular data to voucher specimens and hence, georeferenced populations when available.

These 104 individuals used in the phylogenetic analysis included 64 putative Sri Lankan species 4 Indian species. (S1 Table). This dataset included 31 unnamed putative species (marked ‘cf.’ or ‘sp.’) for which molecular data were available and could be verified through voucher numbers as well [2]. To serve as the outgroup, we included two species of *Raorchestes*, a putative sister group of *Pseudophilautus* [2]. All available nominal species of Sri Lankan *Pseudophilautus* were included, to achieve the best possible taxon sampling. We followed nomenclature by the Amphibian Species of the World 6.0 online reference of the American Museum of Natural History (https://amphibiansoftheworld.amnh.org/index.php) [5] and the voucher numbers provided in the taxonomic literature in recognizing the species of *Pseudophilautus*. Frost, 2020 [5] catalogues a total 80 species of *Pseudophilautus*, out of which 17 are extinct [3, 16, 19]. Among the extant taxa, 10 lack molecular sequence data in Genbank, yielding a total of 53 extant species with molecular sequence data. These 53 include four Indian species and 49 Sri Lankan species.

### Phylogenetic analyses

Initially, to infer the phylogenetic relationships among *Pseudophilautus* and to use as a guide tree, we constructed a multi gene phylogeny using 16S rRNA, 12S rRNA and Rag-1 molecular loci. Mitochondrial 16S rRNA and 12S rRNA gene fragments were aligned using MUSCLE as implemented by MEGA v.6.0 [42]; regions for which we had low confidence in positional homology were removed from the analysis. Nuclear Rag-1 gene sequences were aligned using MEGA v.6.0 with translated amino-acid sequences. The complete concatenated dataset included 106 taxa with a total of 2189 bp. Nucleotide composition of each gene fragment is provided in S2 Table.

Tree topology was inferred and the posterior probability at each node was assessed using a Bayesian statistical framework using BEAST v.1.4 [43] that was performed for both partitioned and unpartitioned datasets as well as the individual gene fragments. The dataset was partitioned into specific gene regions by specifying character sets (charset 16S rRNA = 1–481; charset 12S rRNA = 482–795; charset Rag-1 = 796–2189). The partitioned dataset was used for the phylogenetic analyses discussed in detail. The best-fitting nucleotide substitution model for each dataset was chosen using jModelTest v.2.1.4 [44, 45]. Model GTR+I+G as the nucleotide substitution model, Yule model as the tree prior; and lognormal relaxed clock as the molecular clock were assigned in BEAST and the analysis was run in Cipres Science Gateway Server [46] for 50 million generations and for two consecutive runs. Burnin was defined by observing the log-output file in Tracer v.1.6 [47]; 90% of the post-burnin trees were analyzed using Tree Annotator and a final maximum clade credibility tree was constructed. Similarly, separate gene trees were constructed using 16S rRNA, 12S rRNA and Rag-1 gene fragments and their topologies inferred. Subsequently, to establish consistency among trees, the un-partitioned dataset and the three genes individually were also analysed using RAxML.

### Assessing species boundaries

Due to scarcity of available molecular sequence data, we were in some cases unable to include more than one sequence per species in our dataset. Although introducing singletons could potentially bias the analyses, studies have shown that methods such as ABGD perform well with singletons [48]; further, increasing the number of loci and the sample size per species does not have a significant impact on the species delimitation results [13]. However, multiple authors have cautioned against using single individuals per species and a single locus for species in delimitation studies [49, 50]. Using indiscriminate divergence thresholds to delimit species can result in over- or underestimation of species diversity as there is no universally accepted threshold to distinguish intra- from interspecific genetic divergence when using data sets involving singletons or single loci [10]. Therefore, to define species boundaries specifically for species delimitation containing singletons, obtaining different species-delimitation schemes over a range of prior intraspecific divergence limits to assess the consistency of divergence thresholds [10], testing with multiple methods as well as multiple genes and looking for congruence amongst them need to be considered [51]. Nevertheless, one should always consider the results of any of the methods used with caution and cross-compare them with results obtained using other approaches before reaching conclusions [52].

To accomplish this, for rapid and large-scale assessment of species limits using a single locus, we implemented four methods covering distance and tree-based methods: Automatic Barcode Gap Discovery (ABGD) method [34], Bayesian Poisson Tree Processes model (bPTP) [37], Multi-rate Poisson tree processes for single-locus species (mPTP) [38] and the Bayesian version of the general mixed yule-coalescent model (bGMYC) [53]. We specifically applied this method to the 16S rRNA gene fragment (520 bp), a universal DNA barcoding marker for amphibians, which is being widely used to delimit species [10, 22]. Further, it is the most widely available gene fragment for all the taxa considered in this analysis [22, 23]. Similarly, we carried out these analyses for 12S rRNA, Rag-1 nDNA as well as the concatenated data set for all three gene fragments. We also performed the above analyses for data sets containing only singletons to cross-compare and check if having only singletons would affect the final outcome.

Initially, pairwise genetic distances were calculated among all species for all loci as well as the concatenated data set using PAUP4 [54]. Estimates of evolutionary divergences over sequence pairs within clades were calculated using MEGA X phylogenetic software after removing all ambiguous positions for each sequence pair [55].

In the ABGD method, pairwise genetic distances were calculated using the simple distance model as well as the Kimura (K80) distance model with a transition/transversion rate of 1.5 [10]. For the range of prior intraspecific divergences (P), we sampled 100 values from Pmin = 0.001 to Pmax = 0.1. To obtain a wide range of partitions from conservative to liberal species-delimitation schemes, we used a small relative gap width of X = 0.1. This analysis was performed on the ABGD webserver platform available at http://wwwabi.snv.jussieu.fr/public/abgd/abgdweb.html.

In the bPTP method, the resulting maximum clade credibility trees obtained for the 16S rRNA, 12S rRNA, Rag-1 nDNA gene fragments and the aligned concatenated data set from BEAST was used separately, and the outgroup taxa were defined and removed from the analysis to improve the delimitation results. The MCMC chain was run for five million generations; thinning and burn-in were defined as 100 and 0.1, respectively, and performed on the bPTP webserver platform available at https://species.h-its.org/ptp. The results were checked for convergence, and the resulting species delimitations were compared with other methods used. In the mPTP method, the same Maximum clade credibility tree was used. To obtain the species delimitations, default optimization parameters were run in the mPTP webserver platform available at https://mptp.h-its.org.

Considering that a single-point estimate of a phylogeny is typically associated with substantial uncertainty, hence influencing its accuracy, the bGMYC method implemented in R was used to delimit species. All gene alignments were run in BEAST v.1.4 [42] under HKY nucleotide substitution model using strict clock as well as relaxed clock models and GTR nucleotide substitution model with strict and relaxed clock models. To account for uncertainty, 1000 post burn-in trees sampled from the posterior distribution of ultrametic trees were used with a conspecificity probability thresholds ranging from 0.95–0.98.

When evaluating the possible synonymy of species based on the results of these four methods, we use a conservative approach given that the *Pseudophilautus* and rhacophorids in general are known to evolve slowly [2]. Even species separated by modest genetic divergences may have evolved over long periods, accumulating changes that are important in reproductive isolation in other dimensions, such as vocalization and morphology, which may not be captured by a study involving just a few DNA markers. However, in consideration of the DNA barcoding marker for amphibians (16S rRNA) [22], we call attention to species groups that are recognized to be a single entity by at least three delimitation methods as putative species that need further taxonomic work to be recognized as valid species. Emphasis was given on the congruency of delimitation results of the Rag-1 nDNA marker and the concatenated data set when interpreting the results obtained by the delimitation of 16S rRNA gene fragment and coming into conclusions.

### Geophylogeny

A geophylogeny was constructed to assess the geographical structuring of the Sri Lankan *Pseudophilautus* diversification by building a geophylogeny on GenGIS v. 2.5.3 [56] based on the maximum clade credibility tree. Geographical locations of species were obtained from original species descriptions and IUCN species range maps, and type localities were considered in constructing the geophylogeny. The vouchers that we were unable to georeference accurately were excluded from our geophylogeny. However, in the current analysis, we do not consider geographic distribution to be a criterion for species delimitation. Nevertheless, their relative geographic isolation was considered when interpreting delimitation results of recovered species.

## Results

**Fig 1:**
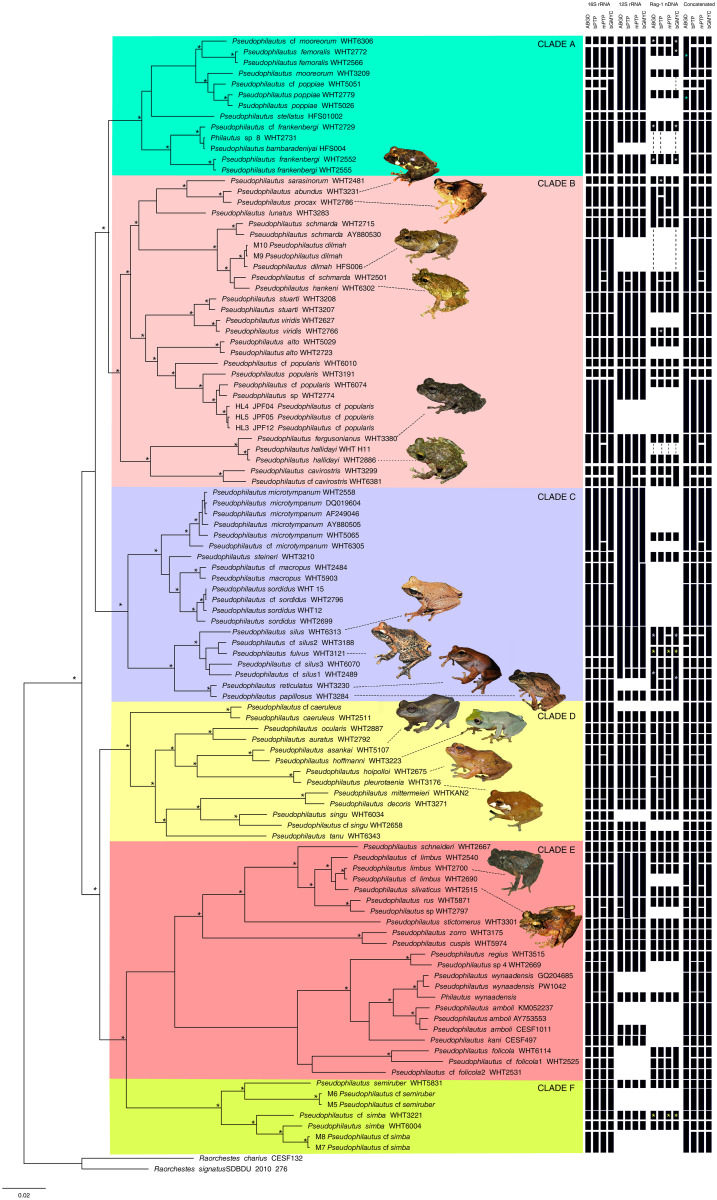
Delimitation summary results. Molecular phylogenetic relationships of *Pseudophilautus*, based on Bayesian inference of the concatenated data set of the 16S rRNA + 12S rRNA + Rag1 (2189bp) loci. Asterisks (*) above nodes represent ≥ 95% Bayesian posterior probabilities. The scale bar represents number of changes per site. Recovered species delimitation based on overall significance of results for molecular species delimitation methods (ABGD, bPTP, mPTP and bGMYC) using 16S rRNA, 12S rRNA, Rag-1 nDNA loci and the concatenated data set are shown as black rectangles on the right. Six major clades of *Pseudophilautus*, A-F, are shaded in different colours on the phylogeny. Images of frogs indicate species suggestive of synonymyzation based on recovered species delimitations. Rectangles indicated by * in similar colours represent species pairs that were recovered as close relatives or recovered in different clades based on their relative position on the concatenated tree.

The phylogenetic analysis indicates that the internal nodes of the *Pseudophilautus* clades are, for the most part, well supported. Building on these relationships, six major clades can be seen in the phylogeny (Fig 1, clades A–F), concordant with the topology recovered by [2]. Topologies of the consensus trees of Bayesian and ML analyses are similar in their assembly of major clades (S1 Fig). Gene trees based on 16S rRNA, 12S rRNA and Rag-1 nDNA are congruent in most instances and clade-level relationships are well established for many taxa (S2 Fig). However, the gene trees have slight differences in their topologies as well; in the 16S tree Clade A is divided into two distantly related clusters and the clade involving *P*. *mooreorum* appears as an early-diverging lineage. In the nuclear Rag-1 tree, the *P*. *fulvus* and *P*. *cuspis* of clade C are more closely related to Clade F while Clade D is recovered as two different clusters (see S2 Fig). These differences were also reflected in the delimitation analyses carried out for different genes.

Pairwise genetic distances among taxa are provided in the S3–S5 Tables. Table 1 show estimates of within clade uncorrected mean phylogenetic distances (p-dist). Overall genetic distances for *Pseudophilautus* are low given their slow accumulation of changes over a long period. Within-clade average distance for 16S rRNA is lowest in Clade C, while the highest average distance is recorded in Clade E.

**Table 1:** Mean within clade uncorrected percent divergences (p-dist) over sequence pairs of mitochondrial 16S rRNA and nuclear Rag-1 gene fragments.

Clade	16S rRNA mtDNA %		Rag-1 nDNA %	
	Range (min—max) %	Average %	Range (min–max)%	Average %
Clade A	1.4–5.6%	3.5%	0.1–1.2%	1%
Clade B	0.2–6.6%	4.6%	0.07–2.6%	1%
Clade C	0.2–4.5%	2.7%	0.2–1.9%	1%
Clade D	0.6–8.9%	5.5%	0.07–3.3%	2%
Clade E	0.4–11.4%	7.2%	0.5–2.7%	2%
Clade F	1.2–4.1%	3.1%	0.9–1.1%	1%

Note that N = 13, 29, 20, 13, 22 and 7 for clades A, B, C, D, E and F, respectively.

In summary, out of 104 *Pseudophilautus* haplotypes, ABGD, bPTP, mPTP and bGMYC recovered a maximum of 60, 61, 67 and 57 potential species, respectively, for the 16S rRNA gene fragment (Fig 1). The ABGD analysis recovered a total of seven partitions with prior maximal intraspecific distances (*p*) ranging from 0.001 to 0.031 and number of delimited species ranging from 1 to 60. The recursive approach recovered a higher number of species compared to the initial run, except partitions one, three and four, which indicated a similar number of species. We selected the partitions that had the smallest maximal intraspecific distances below 0.002 for our interpretations to recover a maximum number of OTUs. Therefore, we use the conservative first partition for discussion, below.

At least three analyses merged the following nominal species: *P*. *procax* and *P*. *abundus* (p-dist = 0.21%); *P*. *hallidayi* and *P*. *fergusonianus* (p-dist = 0.44%); *P*. *reticulatus* and *P*. *papillosus* (p-dist = ~0.00%); *P*. *pleurotaenia* and *P*. *hoipolloi* (p-dist = 0.63%); *P*. *hoffmani* and *P*. *asankai* (p-dist = 1.04%); *P*. *silvaticus* and *P*. *limbus*(p-dist = 0.42%); *P*. *dilmah* and *P*. *hankeni* (p-dist = ~0.69%); *P*. *fulvus* and *P*. *silus* group (p-dist = 1.04%).

The analyses of 16S rRNA gene fragment recovered 8 distinct OTUs from clade A, together with three putative unnamed species (*P*. cf. *frankenbergi* WHT2729, *P*. cf. *mooreorum* WHT6306 and *P*. cf. *poppiae* WHT5051). Although the phylogenetic placement of *P*. *bambaradeniyai* was confirmed in clade A, we did not include it in our final analysis as the sequence identified for that species in Genbank was of doubtful provenance (M. Wickramasinghe, pers. comm.). Clade B contained the greatest number of previously identified taxa (29), of which 18 were recovered as seven clusters by all methods, while *P*. cf. *popularis* WHT6010, *P*. cf. *popularis* WHT6074 and *P*. cf. *cavirostris* WHT6381 were recovered as unidentified species. The highest degree of clustering (~85%) was observed in Clade C, while clade D suggests two potential synonymies (*P*. *pleurotaenia—P*. *hoipolloi*, and *P*. *hoffmanni—P*. *asankai*). About 45% clustering was evident in clade E, which recovered four unidentified species (*P*. cf. *limbus* WHT2540, *Pseudophilautus* sp. 4 WHT2669, *P*. cf. *folicola1* WHT2525 and *P*. cf. *folicola2* WHT2531), whereas clade F recovered *P*. cf. *simba* WHT3221, M5 *P*. cf. *semiruber and* M7 *P*. cf. *semiruber* as putative new species.

In general, the bPTP, mPTP and bGMYC models yielded similar results for the 16S rRNA gene fragment, with a few deviations. While mPTP differentiated *P*. cf. *schmarda* from *P*. *hankeni*—*P*. *dilmah*, other models recovered *P*. *schmarda* as conspecific with *P*. *hankeni*—*P*. *dilmah*. Additionaly, mPTP recovered *P*. *fergusonianus* and *P*. *hallidayi*; *P*. cf. *microtympanum* WHT6305 and *P*. *microtympanum*; *P*. *asankai* and *P*. *hoffmani*; *P*. *rus* and *Pseudophilautus* sp. WHT2797 as distinct OTUs, while all other models recovered them as single OTUs. The bGMYC model identified *P*. cf *poppiae* WHT5051 and *P*. *poppiae*; *P mittermieri* and *P*. *decoris*; *P regius* and *Pseudophilautus sp* WHT2669 as a single OTU, while other models recovered them as distinct. All analyses recovered the previously unnamed *P*. cf. *mooreorum* WHT6306, *P*. cf. *frankenbergi* WHT2729, *P*. cf. *popularis* WHT6010, *P*. cf. *popularis* WHT6074, *P*. cf. *cavirostris* WHT6381, *P*. cf. *silus*1 WHT2489; *P*. cf. *silus*3 WHT6070, *P*. cf. *singu* WHT2658, *P*. cf. *limbus* WHT2540, *P*. cf. *folicola*1 WHT2525, *P*. cf. *folicola*2 WHT2531, M5 *P*. cf. *semiruber*, M7 *P*. cf. *semiruber* and *P*. cf. *simba* WHT3221 as valid OTUs, which may be new species.

However, the delimitation analyses carried out on other loci (12S rRNA and Rag-1 nDNA) as well as the concatenated dataset shows that there are a few incongruencies for some of the species. Compared to 16S rRNA results, Rag-1 nDNA clustered *P*. *reticulatus*, *P*. *pappilosus* and *P*. *silus* together; synonymization of *P*. *limbus* and *P*. *silvaticus* was not supported. It was also unable to recognize *P*. cf. *frankenbergi* WHT2729 as a potential new species. However, lack of Rag-1 nDNA molecular data for many other taxa was a limitation in interpreting most of the results based on 16S rRNA data. The results based on the concatenated data as well as the data comprised only of singletons (S3 Fig) were largely congruent with the delimitations derived from 16S rRNA data.

**Fig 2:**
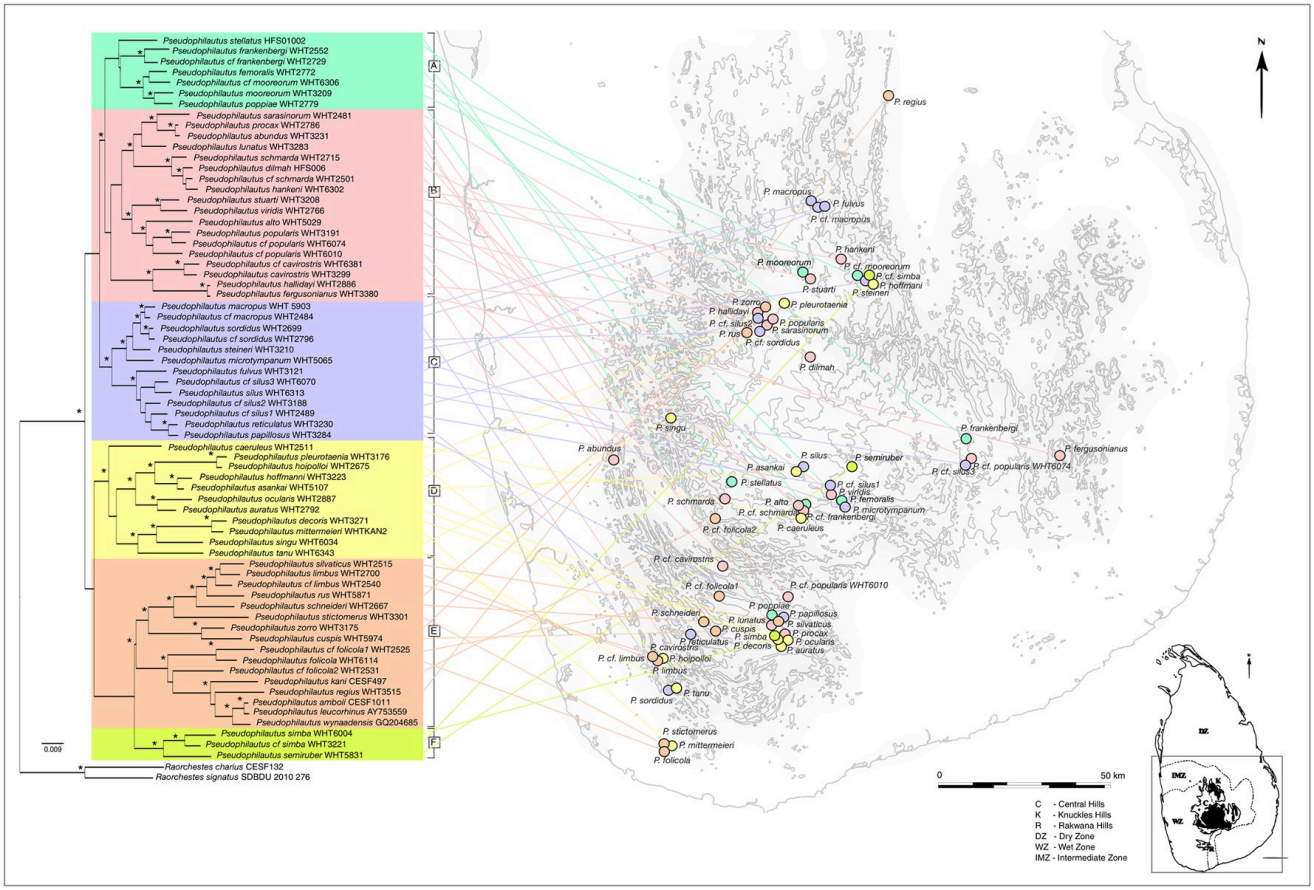
Geophylogeny of Sri Lankan *Pseudophilautus*: The phylogeny of Sri Lankan *Pseudophilautus* laid across the geographic distribution of the species considered in the analysis. Type localities are indicated with circles (see S6 Table for all known locations of *Pseudophilautus*). Different colors represent major clades of *Pseudophilautus* and the three major mountain ranges are highlighted on the overview map of Sri Lanka. A pattern where the six clades identified in the phylogeny, sharing representatives among different geographical regions, is evident. In some of the groups, sister species are distributed across adjacent mountain ranges or from different parts of large montane regions and local species communities are composed of species from disparate clades.

The geophylogeny for the Sri Lankan *Pseudophilautus* diversification revealed complex patterns of geographical structuring (Fig 2). With narrow exceptions, members of the genus are mainly restricted to four distinct geographic regions, namely Central Hills, Knuckles Hills, Rakwana Hills and the Lowland wet zone [2]. However, the six clades identified in the phylogeny occur across these geographic regions. A few clades, however, are altitudinally stratified. Clade A, for example, was restricted to the uplands (>700m asl) while clade E includes mostly lowland (<700m asl) species. In some of the clades, sister species were distributed between adjacent mountain ranges or inhabited different parts of large mountainous regions, though local communities were composed of species from disparate clades.

## Discussion

We tested the species boundaries of Sri Lankan *Pseudophilautus* using single-locus molecular species delimitation methods, which have found wide utility [30, 31, 57, 58]. Methods of molecular species delimitation differ from one another in a number of respects, each with its own limitations [13]. Among the most widely used methods, the generalized mixed Yule-coalescent (GMYC) and Poisson tree processes (PTP) are tree-based methods designed for the analysis of single-locus data but are often applied to concatenations of multilocus data. In contrast, ABGD is a method based on genetic distances computed from a single locus [34]. Generally, due to differences between these methods and their associated issues [40], testing with multiple methods as well as multiple genes and assessing congruence amongst them has been a widely used approach in species delimitation [51]. Nevertheless, the results of any of the methods tested should be compared and interpreted cautiously, integrating also the phenetic and ecological aspects of the concerned species [59].

The four delimitation methods we used for 16S rRNA recovered 57–67 OTUs. These include 14 previously unrecognized OTUs that were unambiguously recovered by all four methods; these may represent undescribed species. Among extant, previously recognized Sri Lankan *Pseudophilautus* (59 species), only 49 were represented by genetic data in the present analysis. Our delimitation criteria applied to the 16S rRNA amphibian barcoding gene suggested that 16 of these 49 nominal species in fact represent eight OTUs. Analyses based on other gene fragments, concatenated data set as well as the data set comprised only of singletons showed low levels of incongruences with the results obtained from 16S data thus strengthening the synonymy of above 16 species.

In general, the rate of evolution of mitochondrial genes is faster than of nuclear genes [60, 61] and this pattern can be seen when 16S rRNA (mtDNA) and 12S rRNA divergences are compared to that of Rag-1 nDNA (S3 and S4 Tables). As expected, the delimitation boundaries for Rag-1 nDNA are stricter than for mtDNA-based analyses, recovering only 42 species from 60 OTUs tested, too conservative to validate, given their morphological diversity.

Subject to verification based also on phenotypic criteria, we therefore propose the following putative synonymies: *Pseudophilautus hallidayi* [15] is likely a synonym of *P*. *fergusonianus* [62]. *Pseudophilautus papillosus* [14] is likely a synonym of *P*. *reticulatus* [63]. *Pseudophilautus hoipolloi* [14] is likely a synonym of *P*. *pelurotaenia* [64]. *Pseudophilautus dilmah* [65] is likely a synonym of *P*. *hankeni* [18]. For the following pairs of putative simultaneous synonyms, we act as first revisers in allocating precedence as follows under Art. 24.2 of the International Code of Zoological Nomenclature (1999): *Pseudophilautus abundus* [14] is likely a synonym of *P*. *procax* [14] (precedence to *P*. *procax*). *Pseudophilautus silvaticus* [14] is likely a synonym of *P*. *limbus* [14] (precedence to *P*. *limbus*). *Pseudophilautus silus* [14] is likely a synonym of *P*. *fulvus* [14] (precedence to *P*. *fulvus*). *Pseudophilautus hoffmani* [15] is likely a synonym of *P*. *asankai* [14] (precedence to *P*. *asankai*).

Therefore, based on the present sampling, the overall species richness of Sri Lankan *Pseudophilautus* would be 65 (33 species unaffected by delimitation + 8 revised species + 14 putative new species + 10 nominal species lacking genetic data, presumed valid). However, the 14 putative new species remain to be assessed phenetically in the context of the general lineage concept of species, where independent evolutionary lineages are recognized using multiple criteria [66]. The criteria that have been used hitherto to test species hypothesis for Sri Lankan *Pseudophilautus* are morphology, mtDNA, geographic distribution and vocalization.

Further, the validity of the remaining ten species lacking genetic data (see S1 Table) too, need to be tested using molecular approaches, as sequences for these species from their topotypes become available. These are *P*. *nemus* [14], *P*. *bambaradeniyai*, *P*. *dayawansai*, *P*. *jagathgunawardanai*, *P*. *karunarathnai*, *P*. *newtonjayawardanei*, *P*. *puranappu*, *P*. *samarakoon*, *P*. *sirilwijesundarai* [20] and *P*. *conniffae* [21].

Although our analyses recovered *P*. *bambaradeniyai* and *P*. cf. *frankenbergi* as a single entity, the original description of *P*. *bambaradeniyayi* shows it to be distinguished from *P*. *frankenbergi* by a relatively smaller body size, a dorsally convex head, a laterally truncated snout, a rounded canthus rostralis, a convex interorbital space, and the absence of fringe on fingers [20]. We therefore consider *P*. *bambaradeniyayi* to be a valid species and consider the Genbank sequence assigned to it (Accession number KP272047) a misidentification. In view of this confusion, we excluded *P*. *bambaradeniyai* from the final analysis due to unavailability of molecular data from a specimen from its type locality.

The geophylogeny highlights the geographic distribution of species pairs which are shown to be discrete OTUs by the delimitation analyses. Contiguity for these species has been established through subsequent field sampling since their original description, where new populations have come to light. Their geophylogeny shows a pattern of sister species distributed across adjacent mountain ranges, or from different parts of large montane regions, highlighting the importance of mountains as a mechanism of isolation and allopatric speciation. Hence, local communities are composed of species from disparate clades that, in most cases, have been assembled through hybridization [67, 68] or migration rather than in situ speciation [2]. Further, lineages could be incompletely isolated for millions of years after their formation. Hence, much evolution of eventual reproductive isolation can occur while nascent species are in gene-flow contact, in sympatry or parapatry, long after divergence begins [67].

Morphological investigations suggest that Clade E is a taxonomically challenging group. This clade comprises of small-bodied species, mainly from the lowland rainforests, which are difficult to distinguish. Further, there are no clear geographic barriers that seem to have isolated its constituent species. Their populations may, however, have been isolated historically but coalesced by migration or changes in the extent of suitable habitat [69]. While this clade contains some species that are difficult to distinguish by morphology alone, it also contains a higher number of species yet to be described.

A common pattern of sister species that are merged (synonymized) seem to be populations leading to a pattern of parapatry, where one of the species is widely distributed with the other being a montane isolate. This is seen between *P*. *abundus* (widespread), *P*. *procax* (montane isolate around Rakwana Hills: 1060m asl); and *P*. *reticulatus* (widespread) and *P*. *papillosus* (montane isolate around Handapan Ella: 1270 m asl). Another pattern is limited geneflow between what appeared initially to be montane isolates (*P*. *hoffmanni* and *P*. *asankai*), but later, populations were discovered in valleys connecting these mountains. The distribution of these species fits a pattern of parapatric speciation through limited gene flow.

In clade B, the grouping of the clade [*P*. *hankeni*, *P*. cf. *schmarda* and *P*. *dilmah*] suggests a possible pattern of parapatric speciation through restricted gene flow. Here, *P*. cf. *schmarda* and *P*. *dilmah* are distributed at similar elevations in different parts of the central mountains (Fig 2), while *P*. *hankeni* is confined to the adjacent Knuckles mountain range.

Further, the delimitation failure of *P*. *hallidayi* and *P*. *fergusonianus* in clade B is plausible given the marked overlap in their geographic distributions. The previously known distribution of *P*. *hallidayi* ranged from Namunukula to the mid-elevations of the Central Hills (Gadaladeniya and Namunukula), whereas *P*. *fergusonianus* had a wide range spanning Monaragala, Ginigathhena, Laggala, Pallegama, Deniyaya, Pitadeniya, Gannoruwa to Peradeniya, including both the lowlands as well as mid-elevations of the central and Knuckles hills.

*Pseudophilautus* cf. *silus* 2 in clade C seem to be species found at the edge of the distribution range of *P*. *silus* in the Central Hills; this lends confidence to our treating these populations as belonging to a single species. The recovery of *P*. *fulvus* and *P*. *silus* in clade C, as a single OTU, can be understood in the context of limited gene flow occurring among the populations of the two adjacent mountain regions, Knuckles and Central Hills, respectively, which are separated by the deep valley carved by the Mahaweli River.

Our delimitation analysis relies primarily on the 16s rRNA gene fragment, which is the most widely used barcoding marker in frogs [22]. However, future genetic sampling will help us better understand these frogs. The present framework can be used to develop and test hypotheses on distribution and speciation in *Pseudophilautus*.

Further, it is useful to have a standardized molecular benchmark for delimiting species for this group, in order to objectively validate species. Among the synonymized species the highest uncorrected p-distance was recovered among *P*. *hoffmani* and *P*. *asankai* (p-dist = 1.04%) for the 16S rRNA amphibian barcoding gene. This is perhaps close to the 16S threshold for pairwise species differentiation. Therefore, we propose an uncorrected 16S pairwise distance of 1.5% as a barcoding benchmark for species boundaries in new-species descriptions for *Pseudophilautus*, and also perhaps for rhacophorids in general.

Ideally, however, we suggest testing species boundaries using combinations of delimitation methods and criteria in the context of the PSC. We recommend that future new-species diagnoses and descriptions be based on multiple criteria, including both molecular and morphological approaches, in the context of the general lineage concept [66]. Clearly, at least some of the new synonymies we report here derive from original descriptions based on small sample size, highly localized sampling or polymorphic species. Integrative approaches may help avoid the creation of unnecessary names in future. Testing species boundaries using the general lineage concept will also help recognize young species which are separated by modest molecular divergences but which are nevertheless independent evolutionary entities.

Molecular species delimitation is a useful tool to refine the assessment of species hypotheses, which here we test in the case of Sri Lankan *Pseudophilautus*. For almost all species assessed using the IUCN Red List Criteria, it is criterion B (restricted distribution of a species), where Extent of Occurrence (the area contained within the shortest continuous imaginary boundary that can be drawn to encompass all the known, inferred or projected sites of occurrence, excluding cases of vagrancy) and Area of Occupancy (the area within its Extent of Occurrence which is occupied by a taxon, excluding cases of vagrancy) plays a major role in the assignment of threat categories, which lead to prioritization for conservation. Hence, a clearer understanding of operational taxonomic units provides a framework for targeted conservation action.

## Supporting information

S1 Table Details of the GenBank specimens used in the molecular analyses, with their voucher references and GenBank accession numbers. (DOCX)

S2 Table Nucleotide composition for mitochondrial 16S, 12S and nuclear Rag1 gene fragments of each taxon used in the analyses. (DOCX)

S3 Table Uncorrected pairwise genetic distances among taxa of the genus *Pseudophilautus* for 16S rRNA gene fragment. (DOCX)

S4 Table Uncorrected pairwise genetic distances among taxa of the genus *Pseudophilautus* for Rag -1 nDNA gene fragment. (DOCX)

S5 Table Uncorrected pairwise genetic distances among taxa of the genus *Pseudophilautus* for concatenated 16S+12S+Rag1 sequences. (DOCX)

S6 Table Geographical locations of all species of Sri Lankan *Pseudophilautus* indicated in Fig 2. (DOCX)

S1 Fig Molecular phylogenetic relationship of *Pseudophilautus*, based on maximum likelihood inference of the 16S rRNA + 12S rRNA and Rag1 concatenated data set. (PDF)

S2 Fig Molecular phylogenetic relationship of *Pseudophilautus*, based on Bayesian inference of the 16S rRNA (A), 12S rRNA (B) and Rag1 (C) gene fragments. (PDF)

S3 Fig Recovered species delimitation based on overall significance of results for molecular species delimitation methods (ABGD, bPTP, mPTP and bGMYC) using 16S rRNA, 12S rRNA, Rag-1 nDNA and concatenated data set on only singletons. (PDF)
