# Correction: Molecular species delimitation of shrub frogs of the genus *Pseudophilautus* (Anura, Rhacophoridae)

**DOI:** 10.1371/journal.pone.0306069

**Published:** 2024-06-21

**Authors:** Gajaba Ellepola, Jayampathi Herath, Kelum Manamendra-Arachchi, Nayana Wijayathilaka, Gayani Senevirathne, Rohan Pethiyagoda, Madhava Meegaskumbura

## Notice of Republication

An incorrect version of Fig 1 was published in error. This article was republished on June 5, 2024, to correct the error. Please download the article again to view the correct version.
