# Supplementary material for: Molecular species delimitation of shrub frogs of the genus Pseudophilautus (Anura, Rhacophoridae)
Source: PLoS One. 2021 Oct 19;16(10):e0258594. doi: 10.1371/journal.pone.0258594 (PMC8525734; doi:10.1371/journal.pone.0258594)
Supplement: S1 Table — (DOCX) [file pone.0258594.s001.docx]

S1 Table: Details of the GenBank specimens used in the molecular analyses, with their voucher references and GenBank accession numbers. Morpho-species which lack genetic data are also provided in the table.

| **Species** | **Voucher number** | **Accession number** | | |
| --- | --- | --- | --- | --- |
|  |  | **16S RNA** | **12S RNA** | **Rag-1** |
| *Pseudophilautus abundus* | WHT3231 | MH789433 | MK020187 | MK007029 |
| *Pseudophilautus adspersus* | BMNH 1947.2.6.23 | - | - | - |
| *Pseudophilautus alto* | WHT5029 | GQ204677 | GQ204738 | GQ204562 |
| *Pseudophilautus alto* | WHT2723 | AY141827 | AY141781 | - |
| *Pseudophilautus amboli* | CESF1011 | JX092658 | JX092727 | - |
| *Pseudophilautus amboli* | PA1205 | KM052237 |  |  |
| *Pseudophilautus amboli* | AY75355559 | AY753559 |  |  |
| *Pseudophilautus asankai* | WHT5107 | FJ788160 | FJ788141 | - |
| *Pseudophilautus auratus* | WHT2792 | AY141835 | AY141789 | MK007018 |
| *Pseudophilautus bambaradeniyai* | HFS004 | KP272047 | - | - |
| *Pseudophilautus caeruleus* | WHT2511 | AY141810 | AY141764 | MK007003 |
| *Pseudophilautus cf caeruleus#* | - | - | - | - |
| *Pseudophilautus cavirostris* | WHT3299 | GQ204676 | GQ204737 | GQ204561 |
| *Psudophilautus* cf *cavirostris* | WHT6381 | MH789448 | MK020199 | - |
| *Pseudophilautus* cf *folicola*1 | WHT2525 | AY141812 | AY141766 | - |
| *Pseudophilautus* cf *folicola*2 | WHT2531 | MH789427 | - | - |
| *Pseudophilautus* cf *frankenbergi* | WHT2729 | AY141828 | AY141782 | - |
| *Pseudophilautus* cf *limbus* | WHT2540 | AY141813 | AY141767 | - |
| *Pseudophilautus* cf *limbus* | WHT2690 | AY141823 | AY141777 | - |
| *Pseudophilautus* cf *macropus* | WHT2484 | AY141808 | AY141762 | - |
| *Pseudophilautus* cf *microtympanum* | WHT6305 | MH789445 | MK020196 | - |
| *Pseudophilautus* cf *mooreorum* | WHT6306 | MH789446 | MK020197 | - |
| *Pseudophilautus* cf *poppiae* | WHT5051 | MH789485 | MK020189 | - |
| *Pseudophilautus* cf *popularis#* | HL3JPF12 | - | - | - |
| *Pseudophilautus* cf *popularis#* | HL4JPF04 | - | - | - |
| *Pseudophilautus* cf *popularis#* | HL5JPF05 | - | - | - |
| *Pseudophilautus* cf *popularis* | WHT6010 | MH789439 | MK020193 | - |
| *Pseudophilautus* cf *popularis* | WHT6074 | MH789443 | MK020194 | - |
| *Pseudophilautus cf schmarda* | WHT2501 | MH789426 | MK020182 | - |
| *Pseudophilautus* cf *silus* 1 | WHT2489 | AY141809 | AY141763 | - |
| *Pseudophilautus* cf *silus*2 | WHT3188 | MH789431 | MK020185 | - |
| *Pseudophilautus cf silus3* | WHT6070 | MH789441 | - | - |
| *Pseudophilautus* cf *simba#* | M7 | - | - | - |
| *Pseudophilautus* cf *simba* # | M8 | - | - | - |
| *Pseudophilautus* cf *simba* | WHT3221 | FJ788167 | - | - |
| *Pseudophilautus* cf *singu* | WHT2658 | AY141819 | AY141773 | - |
| *Pseudophilautus* cf *sordidus* | WHT2796 | MH789451 | MK20202 | - |
| *Pseudophilautus* cf *semiruber#* | M5 | - | - | - |
| *Pseudophilautus* cf *semiruber#* | M6 | - | - | - |
| *Pseudophilautus conniffae* | NMSL WCSG 0005 | - | - | - |
| *Pseudophilautus cuspis* | WHT5974 | MH789438 | MK020192 | MK007035 |
| *Pseudophilautus dayawansai* | NMSL 2013.02.01 NH | - | - | - |
| *Pseudophilautus decoris* | WHT3271 | FJ788163 | FJ788144 | MK007030 |
| *Pseudophilautus dilmah* | HFS006 | KP272046 |  |  |
| *Pseudophilautus dilmah#* | M9 |  |  |  |
| *Pseudophilautus dilmah#* | M10 |  |  |  |
| *Pseudophilautus dimbullae* | MCZ A-20878 | - | - | - |
| *Pseudophilautus eximius* | MCZ A-20879 | - | - | - |
| *Pseudophilautus extirpo* | NHBM1236 | - | - | - |
| *Pseudophilautus femoralis* | WHT2772 | AY141831 | AY141785 | MK007016 |
| *Pseudophilautus femoralis* | WHT2779 | AY141833 | AY141787 | GQ204555 |
| *Pseudophilautus fergusonianus* | WHT3380 | AY880501 | AY880586 | MK007044 |
| *Pseudophilautus folicola* | WHT6114 | GQ204680 | X | GQ204564 |
| *Pseudophilautus frankenbergi* | WHT2552 | AY141814 | AY141768 | MK007008 |
| *Pseudophilautus frankenbergi* | WHT2555 | AY141815 | AY141769 | - |
| *Pseudophilautus fulvus* | WHT3121 | MH789430 | MK020184 | MK007020 |
| *Pseudophilautus hallidayi* | WHT2886 | MH789429 | MK020183 | MK007019 |
| *Pseudophilautus hallidayi* | WHT H11 | AY141839 | AY141793 | - |
| *Pseudophilautus halyi* | BMNH 1947.2.6.16 | - | - | - |
| *Pseudophilautus hankeni* | WHT6302 | MH789444 | MK020195 | MK007040 |
| *Pseudophilautus hoffmanni* | WHT3223 | GQ204673 | GQ204736 | GQ204558 |
| *Pseudophilautus hoipolloi* | WHT2675 | AY141822 | AY141776 | MK007012 |
| *Pseudophilautus hypomelas** | NMSL 2013.26.01 NH | - | - | - |
| *Pseudophilautus jagathgunawardanai* | NMSL 2013.03.01 NH | - | - | - |
| *Pseudophilautus kani* | CESF497 | JX092724 | JX092754 | - |
| *Pseudophilautus karunarathnai* | NMSL 2013.04.01 NH | - | - | - |
| *Pseudophilautus leucorhinus* | ZMB3057 | - | - | - |
| *Pseudophilautus limbus* | WHT2700 | GQ204668 | AY141779 | GQ204553 |
| *Pseudophilautus lunatus* | WHT3283 | GQ204675 | FJ788150 | GQ204560 |
| *Pseudophilautus macropus* | WHT5903 | AY141808 | AY141762 | - |
| *Pseudophilautus maia* | BMNH 76.3.21.18 | - | - | - |
| *Pseudophilautus malcolmsmithi* | ZMB 9037 | - | - | - |
| *Pseudophilautus microtympanum* | WHT5065 | GQ204678 | GQ204739 | GQ204563 |
| *Pseudophilautus microtympanum* | AF249046 | AF249046 | - | - |
| *Pseudophilautus microtympanum* | AY880505 | AY880505 | - | - |
| *Pseudophilautus microtympanum* | DQ019604 | DQ019604 | - | - |
| *Pseudophilautus mittermeieri* | WHTKAN2 | GQ204681 | GQ204741 | GQ204565 |
| *Pseudophilautus mooreorum* | WHT3209 | FJ788153 | FJ788134 | MK007025 |
| *Pseudophilautus nanus* | BMNH 1947.2.7.78 | - | - | - |
| *Pseudophilautus nasutus* | BMNH 1947.2.6.21 | - | - | - |
| *Pseudophilautus newtonjayawardanei* | NMSL 2013.05.01 NH | - | - | - |
| *Pseudophilautus ocularis* | WHT2887 | FJ788164 | FJ788145 | - |
| *Pseudophilautus oxyrhynchus* | BMNH 1947.2.6.40 | - | - | - |
| *Pseudophilautus papillosus* | WHT3284 | FJ788170 | FJ788151 | MK007031 |
| *Pseudophilautus pardus* | BMNH 1947.2.7.96 | - | - | - |
| *Pseudophilautus pleurotaenia* | WHT3176 | FJ788165 | FJ788146 | MK007021 |
| *Pseudophilautus poppiae* | WHT2779 | GQ204670 | FJ788136 | GQ204555 |
| *Pseudophilautus poppiae* | WHT5026 | FJ788154 | FJ788135 | - |
| *Pseudophilautus popularis* | WHT3191 | FJ788168 | FJ788149 | MK007023 |
| *Pseudophilautus procax* | WHT2786 | AY141834 | AY141788 | MK007017 |
| *Pseudophilautus puranappu* | NMSL 2013.06.01 NH | - | - | - |
| *Pseudophilautus regius* | WHT3515 | GQ204682 | GQ204742 | GQ204566 |
| *Pseudophilautus reticulatus* | WHT3230 | GQ204674 | - | GQ204559 |
| *Pseudophilautus rugatus* | ZMB8557 | - | - | - |
| *Pseudophilautus rus* | WHT5871 | MH789437 | MK020191 | MK007034 |
| *Pseudophilautus samarakoon* | (NMSL 2013.07.01 NH | - | - | - |
| *Pseudophilautus sarasinorum* | WHT2481 | GQ204667 | AY141761 | GQ204552 |
| *Pseudophilautus schmarda* | WHT2715 | GQ204669 | AY141780 | GQ204554 |
| *Pseudophilautus schmarda* | AY880530 | AY880530 | - | - |
| *Pseudophilautus schneideri* | WHT2667 | AY141820 | AY141774 | MK007010 |
| *Pseudophilautus semiruber* | WHT5831 | MH789436 | MK020190 | MK007033 |
| *Pseudophilautus silus* | WHT6313 | MH789447 | MK020198 | MK007043 |
| *Pseudophilautus silvaticus* | WHT2515 | AY141811 | AY141765 | MK007004 |
| *Pseudophilautus simba* | WHT6004 | GQ204679 | GQ204740 | - |
| *Pseudophilautus singu* | WHT6034 | MH789440 | - | MK007037 |
| *Pseudophilautus sirilwijesundarai* | NMSL 2013.08.01 NH | - | - | - |
| *Pseudophilautus sordidus* | WHT2699 | AY141824 | AY141778 | - |
| *Pseudophilautus sordidus* | WHT H12 | AY141837 | AY141791 | - |
| *Pseudophilautus sordidus* | WHT H15 | AY141838 | AY141792 | - |
| *Pseudophilautus* sp. | WHT2731 | AY141829 | AY141783 | - |
| *Pseudophilautus* sp. | WHT2774 | AY141832 | AY141786 | - |
| *Pseudophilautus* sp. | WHT2797 | AY141836 | AY141790 | - |
| *Pseudophilautus* sp. 4 | WHT2669 | AY141821 | AY141775 | - |
| *Pseudophilautus steineri* | WHT3210 | FJ788157 | FJ788138 | MK007026 |
| *Pseudophilautus stellatus* | HFS01002 | JN862536 | JN862535 | - |
| *Pseudophilautus stictomerus* | WHT3301 | MH789434 | MK020188 | MK007032 |
| *Pseudophilautus stuarti* | WHT3207 | GQ204672 | GQ204735 | GQ204557 |
| *Pseudophilautus stuarti* | WHT3208 | FJ788159 | FJ788140 | - |
| *Pseudophilautus tanu* | WHT6343 | FJ788171 | FJ788152 | - |
| *Pseudophilautus temporalis* | BMNH 1947.2.6.9 | - | - | - |
| *Pseudophilautus variabilis* | BMNH 1947.2.7.87 | - | - | - |
| *Pseudophilautus viridis* | WHT2766 | AY141830 | AY141784 | MK007015 |
| *Pseudophilautus viridis* | WHT2627 | AY141818 | AY141772 | - |
| *Pseudophilautus wynaadensis* | GQ204685 | GQ204685 | GQ204743 | GQ204568 |
| *Pseudophilautus wynaadensis* | 5736 | KJ631353 | - | - |
| *Pseudophilautus wynaadensis* | PW1042 | KM052239 | - | - |
| *Pseudophilautus zal* | BMNH 1947.2.7.94 | - | - | - |
| *Pseudophilautus zimmeri* | ZMB6111 | - | - | - |
| *Pseudophilautus zorro* | WHT3175 | GQ204671 | FJ788147 | GQ204556 |
| *Raorchestes charius* | CESF132 | JX092691 | JX092736 | - |
| *Raorchestes signatus* | SDBDU2010 276 | KU169986 | KU170011 | KU169961 |

Note: Extinct species are indicated in red. Indian species are indicated in blue. Morpho-species which lack genetic data are highlighted. *Species which were thought to be extinct but later have been rediscovered. #Molecular sequences of specimens included in the analyses (based on other ecological studies) which lack GenBank accession numbers.
