# Supplementary material for: Molecular species delimitation of shrub frogs of the genus Pseudophilautus (Anura, Rhacophoridae)
Source: PLoS One. 2021 Oct 19;16(10):e0258594. doi: 10.1371/journal.pone.0258594 (PMC8525734; doi:10.1371/journal.pone.0258594)
Supplement: S2 Table — (DOCX) [file pone.0258594.s002.docx]

**S2 Table. Nucleotide composition for mitochondrial 16S, 12S and nuclear Rag1 gene fragments of each taxon used in the analyses**

| Species | 16S rRNA mtDNA % Nucleotide Composition % | | | |  | 12S rRNA mtDNA %Nucleotide Composition % | | | |  | Rag-1 nDNA Nucleotide Composition % | | | |
| --- | --- | --- | --- | --- | --- | --- | --- | --- | --- | --- | --- | --- | --- | --- |
|  | T(U) | C | A | G |  | T(U) | C | A | G |  | T(U) | C | A | G |
| *Pseudophilautus abundus* WHT3231 | 22.73 | 25.19 | 32.01 | 20.08 |  | 21.97 | 23.57 | 34.39 | 20.06 |  | 24.28 | 22.11 | 30.71 | 22.90 |
| *Pseudophilautus alto* WHT2723 | 23.48 | 24.43 | 31.44 | 20.64 |  | 20.77 | 25.24 | 32.91 | 21.09 |  |  |  |  |  |
| *Pseudophilautus alto* WHT5029 | 23.48 | 24.43 | 31.44 | 20.64 |  | 20.77 | 25.24 | 32.91 | 21.09 |  | 24.23 | 22.28 | 30.57 | 22.93 |
| *Pseudophilautus amboli* CESF1011 | 23.53 | 23.72 | 32.26 | 20.49 |  | 24.00 | 22.67 | 34.67 | 18.67 |  |  |  |  |  |
| *Pseudophilautus amboli* AY753559 | 23.53 | 23.53 | 32.45 | 20.49 |  |  |  |  |  |  |  |  |  |  |
| *Pseudophilautus amboli* KM052237 | 23.53 | 23.72 | 32.26 | 20.49 |  |  |  |  |  |  |  |  |  |  |
| *Pseudophilautus asankai* WHT5107 | 21.56 | 25.00 | 32.63 | 20.80 |  | 22.76 | 24.04 | 32.69 | 20.51 |  | 24.22 | 22.13 | 30.64 | 23.00 |
| *Pseudophilautus auratus* WHT2792 | 21.52 | 25.33 | 32.76 | 20.38 |  |  |  |  |  |  | 23.81 | 22.21 | 30.82 | 23.15 |
| *Pseudophilautus bambaradeniyai* HFS004 | 23.48 | 24.24 | 30.68 | 21.59 |  |  |  |  |  |  |  |  |  |  |
| *Pseudophilautus caeruleus* WHT2511 | 22.92 | 24.24 | 33.14 | 19.70 |  | 20.83 | 25.32 | 33.97 | 19.87 |  | 24.15 | 22.27 | 30.80 | 22.78 |
| *Pseudophilautus cavirostris* WHT3299 | 22.58 | 24.40 | 32.46 | 20.56 |  | 21.73 | 23.64 | 33.55 | 21.09 |  | 24.41 | 21.89 | 31.25 | 22.46 |
| *Pseudophilautus* cf *caeruleus* | 22.92 | 24.24 | 33.14 | 19.70 |  |  |  |  |  |  |  |  |  |  |
| *Pseudophilautus* cf *cavirostris* WHT6381 | 25.00 | 23.62 | 31.30 | 20.08 |  | 22.36 | 23.00 | 33.55 | 21.09 |  | 24.52 | 21.94 | 31.02 | 22.53 |
| *Pseudophilautus* cf *folicola*1 WHT2525 | 23.57 | 23.76 | 31.37 | 21.29 |  |  |  |  |  |  | 24.02 | 22.64 | 30.91 | 22.42 |
| *Pseudophilautus* cf *folicola*2 WHT2531 | 24.62 | 22.35 | 32.01 | 21.02 |  |  |  |  |  |  | 23.94 | 22.64 | 30.64 | 22.78 |
| *Pseudophilautus* cf *frankenbergi* WHT2729 | 23.48 | 24.24 | 30.49 | 21.78 |  | 22.04 | 23.96 | 34.50 | 19.49 |  | 24.42 | 21.80 | 31.18 | 22.60 |
| *Pseudophilautus* cf *limbus* WHT2540 | 23.53 | 23.53 | 32.26 | 20.68 |  | 22.29 | 23.57 | 34.71 | 19.43 |  | 24.07 | 22.49 | 30.46 | 22.99 |
| *Pseudophilautus* cf *limbus* WHT2690 | 23.53 | 23.53 | 32.07 | 20.87 |  | 22.29 | 23.57 | 34.71 | 19.43 |  | 24.13 | 22.47 | 30.49 | 22.90 |
| *Pseudophilautus* cf *macropus* WHT2484 | 23.40 | 24.15 | 32.45 | 20.00 |  | 21.09 | 24.28 | 34.50 | 20.13 |  |  |  |  |  |
| *Pseudophilautus* cf *microtympanum* WHT6305 | 23.25 | 24.20 | 32.14 | 20.42 |  |  |  |  |  |  |  |  |  |  |
| *Pseudophilautus* cf *mooreorum* WHT6306 | 23.86 | 23.86 | 31.44 | 20.83 |  |  |  |  |  |  | 24.31 | 21.87 | 31.21 | 22.61 |
| *Pseudophilautus* cf *poppiae* WHT5051 | 23.77 | 23.96 | 31.32 | 20.94 |  |  |  |  |  |  |  |  |  |  |
| *Pseudophilautus* cf *popularis* HL3 JPF12 | 23.48 | 24.43 | 31.63 | 20.45 |  |  |  |  |  |  |  |  |  |  |
| *Pseudophilautus* cf *popularis* HL4 JPF04 | 23.48 | 24.43 | 31.63 | 20.45 |  |  |  |  |  |  |  |  |  |  |
| *Pseudophilautus* cf *popularis* HL5 JPF05 | 24.24 | 23.86 | 32.01 | 19.89 |  |  |  |  |  |  |  |  |  |  |
| *Pseudophilautus* cf *popularis* WHT6010 | 23.48 | 24.43 | 31.63 | 20.45 |  | 21.09 | 25.56 | 32.91 | 20.45 |  | 23.91 | 22.59 | 30.69 | 22.81 |
| *Pseudophilautus* cf *popularis* WHT6074 | 24.24 | 23.86 | 32.01 | 19.89 |  | 20.77 | 25.24 | 34.19 | 19.81 |  | 23.37 | 23.15 | 30.62 | 22.86 |
| *Pseudophilautus* cf *schmarda* WHT2501 | 22.92 | 24.43 | 32.01 | 20.64 |  |  |  |  |  |  | 24.08 | 22.47 | 30.67 | 22.78 |
| *Pseudophilautus* cf *silus*1 WHT2489 | 23.11 | 24.62 | 31.82 | 20.45 |  | 21.97 | 23.57 | 34.71 | 19.75 |  | 24.24 | 22.22 | 30.95 | 22.58 |
| *Pseudophilautus* cf *silus*2 WHT3188 | 23.67 | 24.05 | 32.20 | 20.08 |  | 21.97 | 23.57 | 34.39 | 20.06 |  | 23.92 | 22.54 | 30.71 | 22.83 |
| *Pseudophilautus* cf *silus*3 WHT6070 | 23.67 | 24.24 | 31.82 | 20.27 |  |  |  |  |  |  | 24.03 | 22.16 | 31.08 | 22.73 |
| *Pseudophilautus* cf *simba* M7 | 24.29 | 22.77 | 32.64 | 20.30 |  |  |  |  |  |  |  |  |  |  |
| *Pseudophilautus* cf *simba* M8 | 24.29 | 22.77 | 32.64 | 20.30 |  |  |  |  |  |  |  |  |  |  |
| *Pseudophilautus* cf *simba* WHT3221 | 24.29 | 22.77 | 32.64 | 20.30 |  |  |  |  |  |  | 24.28 | 22.11 | 30.71 | 22.90 |
| *Pseudophilautus* cf *singu* WHT2658 | 23.62 | 23.81 | 32.38 | 20.19 |  | 21.66 | 24.20 | 33.44 | 20.70 |  |  |  |  |  |
| *Pseudophilautus* cf *sordidus* WHT2796 | 22.92 | 24.62 | 32.01 | 20.45 |  |  |  |  |  |  |  |  |  |  |
| *Pseudophilautus* cf. *semiruber* M5 | 22.62 | 23.76 | 32.51 | 21.10 |  |  |  |  |  |  |  |  |  |  |
| *Pseudophilautus* cf. *semiruber* M6 | 22.62 | 23.76 | 32.51 | 21.10 |  |  |  |  |  |  |  |  |  |  |
| *Pseudophilautus cuspis* WHT5974 | 23.15 | 24.86 | 31.69 | 20.30 |  | 21.66 | 24.20 | 32.48 | 21.66 |  | 24.30 | 22.21 | 31.11 | 22.37 |
| *Pseudophilautus decoris* WHT3271 | 23.72 | 24.29 | 32.45 | 19.54 |  | 24.52 | 21.94 | 33.23 | 20.32 |  | 24.53 | 22.26 | 31.32 | 21.89 |
| *Pseudophilautus dilmah* HFS006 | 22.77 | 24.48 | 31.50 | 21.25 |  |  |  |  |  |  |  |  |  |  |
| *Pseudophilautus dilmah* M10 | 22.77 | 24.48 | 31.50 | 21.25 |  |  |  |  |  |  |  |  |  |  |
| *Pseudophilautus dilmah* M9 | 22.77 | 24.48 | 31.50 | 21.25 |  |  |  |  |  |  |  |  |  |  |
| *Pseudophilautus femoralis* WHT2772 | 23.44 | 24.20 | 31.76 | 20.60 |  | 22.29 | 23.25 | 33.76 | 20.70 |  | 24.29 | 22.32 | 30.78 | 22.61 |
| *Pseudophilautus femoralis* WHT2779 | 24.15 | 23.21 | 31.89 | 20.75 |  | 22.04 | 23.64 | 33.87 | 20.45 |  |  |  |  |  |
| *Pseudophilautus fergusonianus* WHT3380 | 22.73 | 25.00 | 31.82 | 20.45 |  | 22.36 | 23.00 | 33.55 | 21.09 |  |  |  |  |  |
| *Pseudophilautus folicola* WHT6114 | 23.53 | 23.53 | 33.02 | 19.92 |  |  |  |  |  |  | 24.07 | 22.25 | 30.84 | 22.84 |
| *Pseudophilautus frankenbergi* WHT2552 | 23.34 | 24.48 | 30.93 | 21.25 |  | 21.02 | 24.52 | 33.76 | 20.70 |  | 24.37 | 22.13 | 31.02 | 22.49 |
| *Pseudophilautus frankenbergi* WHT2555 | 23.34 | 24.48 | 30.93 | 21.25 |  | 21.02 | 24.52 | 33.76 | 20.70 |  |  |  |  |  |
| *Pseudophilautus fulvus* WHT3121 | 23.48 | 24.24 | 32.20 | 20.08 |  | 21.97 | 23.57 | 34.39 | 20.06 |  | 24.28 | 22.21 | 30.55 | 22.95 |
| *Pseudophilautus hallidayi* WHT11 | 22.20 | 25.43 | 31.31 | 21.06 |  | 22.68 | 23.96 | 32.91 | 20.45 |  |  |  |  |  |
| *Pseudophilautus hallidayi* WHT2886 | 22.20 | 25.43 | 31.31 | 21.06 |  | 22.68 | 23.96 | 32.91 | 20.45 |  | 24.35 | 22.05 | 31.32 | 22.27 |
| *Pseudophilautus hankeni* WHT6302 | 23.11 | 24.43 | 31.44 | 21.02 |  | 21.02 | 25.16 | 32.48 | 21.34 |  | 23.73 | 22.77 | 30.51 | 22.99 |
| *Pseudophilautus hoffmanni* WHT3223 | 21.52 | 25.14 | 32.38 | 20.95 |  | 23.00 | 23.64 | 32.91 | 20.45 |  | 24.33 | 22.17 | 30.53 | 22.97 |
| *Pseudophilautus hoipolloi* WHT2675 | 21.76 | 25.57 | 32.25 | 20.42 |  | 22.44 | 23.08 | 33.33 | 21.15 |  | 24.40 | 22.09 | 30.61 | 22.89 |
| *Pseudophilautus kani* CESF497 | 22.72 | 23.30 | 32.43 | 21.55 |  | 23.33 | 23.33 | 34.33 | 19.00 |  |  |  |  |  |
| *Pseudophilautus limbus* WHT2700 | 23.53 | 23.53 | 32.07 | 20.87 |  | 22.29 | 23.57 | 34.71 | 19.43 |  | 24.07 | 22.49 | 30.46 | 22.99 |
| *Pseudophilautus lunatus* WHT3283 | 22.73 | 25.00 | 31.44 | 20.83 |  | 22.29 | 23.57 | 33.12 | 21.02 |  | 24.26 | 22.30 | 30.92 | 22.52 |
| *Pseudophilautus macropus* WHT5903 | 21.10 | 24.38 | 31.78 | 22.74 |  | 21.05 | 24.56 | 34.04 | 20.35 |  |  |  |  |  |
| *Pseudophilautus microtympanum* AF249046 | 22.68 | 24.57 | 32.33 | 20.42 |  | 21.11 | 24.22 | 35.29 | 19.38 |  |  |  |  |  |
| *Pseudophilautus microtympanum* AY880505 | 22.71 | 23.85 | 33.49 | 19.95 |  | 21.11 | 24.22 | 35.29 | 19.38 |  |  |  |  |  |
| *Pseudophilautus microtympanum* DQ019604 | 22.73 | 24.62 | 32.20 | 20.45 |  | 21.11 | 24.22 | 35.29 | 19.38 |  |  |  |  |  |
| *Pseudophilautus microtympanum* WHT5065 | 22.73 | 24.43 | 32.39 | 20.45 |  | 21.11 | 24.22 | 35.29 | 19.38 |  | 24.32 | 22.09 | 30.94 | 22.66 |
| *Pseudophilautus mittermeieri* WHTKAN2 | 23.72 | 24.29 | 32.26 | 19.73 |  | 23.55 | 22.90 | 32.90 | 20.65 |  | 24.22 | 21.98 | 31.38 | 22.42 |
| *Pseudophilautus mooreorum* WHT3209 | 23.48 | 24.24 | 32.01 | 20.27 |  | 21.68 | 23.78 | 34.62 | 19.93 |  | 24.10 | 22.23 | 31.15 | 22.52 |
| *Pseudophilautus ocularis* WHT2887 | 22.14 | 25.19 | 31.68 | 20.99 |  | 21.41 | 24.92 | 33.23 | 20.45 |  | 23.94 | 22.27 | 31.08 | 22.71 |
| *Pseudophilautus papillosus* WHT3284 | 22.81 | 24.71 | 31.37 | 21.10 |  | 21.73 | 23.64 | 34.50 | 20.13 |  | 24.22 | 22.25 | 30.93 | 22.61 |
| *Pseudophilautus pleurotaenia* WHT3176 | 21.95 | 25.38 | 31.87 | 20.80 |  | 22.44 | 23.40 | 33.01 | 21.15 |  | 24.75 | 21.65 | 30.72 | 22.88 |
| *Pseudophilautus poppiae* WHT2779 | 24.15 | 23.21 | 31.89 | 20.75 |  | 22.04 | 23.64 | 33.87 | 20.45 |  | 24.22 | 21.98 | 30.95 | 22.85 |
| *Pseudophilautus poppiae* WHT5026 | 24.15 | 23.40 | 31.89 | 20.57 |  | 22.04 | 23.64 | 33.87 | 20.45 |  |  |  |  |  |
| *Pseudophilautus popularis* WHT3191 | 23.48 | 24.43 | 32.01 | 20.08 |  | 21.73 | 24.28 | 34.19 | 19.81 |  | 23.82 | 22.81 | 30.63 | 22.74 |
| *Pseudophilautus procax* WHT2786 | 22.92 | 25.00 | 31.82 | 20.27 |  | 21.97 | 23.57 | 34.39 | 20.06 |  | 23.75 | 22.43 | 31.38 | 22.43 |
| *Pseudophilautus regius* WHT3515 | 22.92 | 23.86 | 31.63 | 21.59 |  | 24.60 | 21.73 | 34.50 | 19.17 |  | 24.17 | 22.65 | 30.68 | 22.50 |
| *Pseudophilautus reticulatus* WHT3230 | 22.86 | 24.76 | 31.43 | 20.95 |  |  |  |  |  |  | 24.28 | 22.05 | 30.91 | 22.77 |
| *Pseudophilautus rus* WHT5871 | 24.10 | 22.77 | 32.45 | 20.68 |  | 21.66 | 23.89 | 35.03 | 19.43 |  | 24.39 | 22.59 | 30.43 | 22.59 |
| *Pseudophilautus sarasinorum* WHT2481 | 22.96 | 24.48 | 31.88 | 20.68 |  | 21.41 | 23.64 | 34.50 | 20.45 |  | 24.58 | 22.07 | 31.05 | 22.30 |
| *Pseudophilautus schmarda* AY880530 | 22.97 | 23.78 | 32.11 | 21.14 |  | 21.66 | 24.20 | 32.48 | 21.66 |  |  |  |  |  |
| *Pseudophilautus schmarda* WHT2715 | 23.63 | 23.63 | 31.57 | 21.17 |  | 21.99 | 24.40 | 33.68 | 19.93 |  | 23.82 | 22.73 | 30.79 | 22.66 |
| *Pseudophilautus schneideri* WHT2667 | 24.52 | 23.00 | 32.51 | 19.96 |  | 21.34 | 24.84 | 34.08 | 19.75 |  | 24.24 | 22.42 | 30.70 | 22.64 |
| *Pseudophilautus semiruber* WHT5831 | 22.62 | 23.76 | 32.51 | 21.10 |  | 22.36 | 23.64 | 33.23 | 20.77 |  | 24.15 | 22.34 | 30.75 | 22.77 |
| *Pseudophilautus silus* WHT6313 | 24.05 | 23.67 | 31.63 | 20.64 |  | 21.34 | 23.89 | 34.71 | 20.06 |  | 24.10 | 22.20 | 30.92 | 22.78 |
| *Pseudophilautus silvaticus* WHT2515 | 23.57 | 23.57 | 31.94 | 20.91 |  | 22.29 | 23.25 | 34.71 | 19.75 |  | 23.91 | 22.68 | 30.72 | 22.68 |
| *Pseudophilautus simba* WHT6004 | 24.00 | 23.24 | 32.57 | 20.19 |  | 21.02 | 24.84 | 33.76 | 20.38 |  |  |  |  |  |
| *Pseudophilautus singu* WHT6034 | 23.62 | 23.81 | 32.38 | 20.19 |  |  |  |  |  |  | 24.55 | 21.82 | 31.01 | 22.61 |
| *Pseudophilautus sordidus* WHT12 | 23.06 | 24.39 | 31.76 | 20.79 |  | 20.77 | 24.60 | 34.50 | 20.13 |  |  |  |  |  |
| *Pseudophilautus sordidus* WHT15 | 23.06 | 24.39 | 31.76 | 20.79 |  | 20.77 | 24.60 | 34.50 | 20.13 |  |  |  |  |  |
| *Pseudophilautus sordidus* WHT2699 | 23.06 | 24.39 | 31.76 | 20.79 |  | 20.77 | 24.60 | 34.50 | 20.13 |  |  |  |  |  |
| *Pseudophilautus* sp. WHT2731 | 23.34 | 24.48 | 30.93 | 21.25 |  | 21.02 | 24.52 | 33.76 | 20.70 |  |  |  |  |  |
| *Pseudophilautus* sp. WHT2774 | 24.24 | 23.86 | 32.01 | 19.89 |  | 20.77 | 25.24 | 34.19 | 19.81 |  |  |  |  |  |
| *Pseudophilautus* sp. WHT2797 | 24.10 | 22.77 | 32.45 | 20.68 |  | 21.66 | 23.89 | 35.03 | 19.43 |  |  |  |  |  |
| *Pseudophilautus* sp.4 WHT2669 | 22.92 | 23.86 | 31.63 | 21.59 |  | 24.60 | 21.73 | 34.50 | 19.17 |  |  |  |  |  |
| *Pseudophilautus steineri* WHT3210 | 23.06 | 24.39 | 32.33 | 20.23 |  | 21.15 | 24.04 | 33.97 | 20.83 |  | 23.87 | 22.71 | 30.71 | 22.71 |
| *Pseudophilautus stellatus* HFS01002 | 22.50 | 24.76 | 31.95 | 20.79 |  | 20.77 | 25.00 | 34.15 | 20.07 |  |  |  |  |  |
| *Pseudophilautus stictomerus* WHT3301 | 24.05 | 23.67 | 32.58 | 19.70 |  | 21.73 | 23.64 | 35.14 | 19.49 |  | 24.50 | 22.41 | 30.46 | 22.63 |
| *Pseudophilautus stuarti* WHT3207 | 22.73 | 24.62 | 31.82 | 20.83 |  | 21.15 | 25.00 | 32.69 | 21.15 |  | 24.29 | 22.12 | 31.11 | 22.48 |
| *Pseudophilautus stuarti* WHT3208 | 22.73 | 24.62 | 31.82 | 20.83 |  | 21.15 | 25.00 | 32.69 | 21.15 |  |  |  |  |  |
| *Pseudophilautus tanu* WHT6343 | 22.43 | 24.33 | 33.27 | 19.96 |  | 21.54 | 23.47 | 34.73 | 20.26 |  | 23.89 | 22.42 | 31.05 | 22.64 |
| *Pseudophilautus viridis* WHT2627 | 22.35 | 25.19 | 30.87 | 21.59 |  | 21.09 | 24.92 | 32.91 | 21.09 |  |  |  |  |  |
| *Pseudophilautus viridis* WHT2766 | 22.35 | 25.19 | 30.87 | 21.59 |  | 21.09 | 24.92 | 32.91 | 21.09 |  | 24.50 | 22.26 | 31.05 | 22.19 |
| *Pseudophilautus wynaadensis* | 23.70 | 23.08 | 32.02 | 21.21 |  | 24.00 | 22.67 | 34.67 | 18.67 |  | 24.15 | 22.34 | 31.02 | 22.49 |
| *Pseudophilautus wynaadensis* GQ204685 | 23.34 | 23.53 | 32.07 | 21.06 |  |  |  |  |  |  | 24.15 | 22.34 | 31.02 | 22.49 |
| *Pseudophilautus wynaadensis* PW1042 | 23.70 | 23.08 | 32.02 | 21.21 |  |  |  |  |  |  |  |  |  |  |
| *Pseudophilautus zorro* WHT3175 | 23.38 | 24.71 | 30.80 | 21.10 |  | 22.29 | 23.57 | 32.48 | 21.66 |  | 24.12 | 22.67 | 30.54 | 22.67 |
| Avg. | 23.19 | 24.18 | 31.98 | 20.64 |  | 21.96 | 23.90 | 33.82 | 20.32 |  | 24.18 | 22.30 | 30.85 | 22.67 |
