# Supplementary material for: Molecular species delimitation of shrub frogs of the genus Pseudophilautus (Anura, Rhacophoridae)
Source: PLoS One. 2021 Oct 19;16(10):e0258594. doi: 10.1371/journal.pone.0258594 (PMC8525734; doi:10.1371/journal.pone.0258594)
Supplement: S4 Table — (DOCX) [file pone.0258594.s004.docx]

**S4 Table. Uncorrected Pairwise genetic distances among taxa of the genus Pseudophilautus for Rag-1 nDNA gene fragment**
