# Supplementary material for: Molecular species delimitation of shrub frogs of the genus Pseudophilautus (Anura, Rhacophoridae)
Source: PLoS One. 2021 Oct 19;16(10):e0258594. doi: 10.1371/journal.pone.0258594 (PMC8525734; doi:10.1371/journal.pone.0258594)
Supplement: S6 Table — (DOCX) [file pone.0258594.s006.docx]

S6 Table. Geographical locations of all species of Sri Lankan Pseudophilautus indicated in Figure2. Note that the names of places, the elevation range in which they are recorded and the clade they belong to are provided. Type localities are indicated with bold letters.
