# Supplementary material for: Molecular species delimitation of shrub frogs of the genus Pseudophilautus (Anura, Rhacophoridae)
Source: PLoS One. 2021 Oct 19;16(10):e0258594. doi: 10.1371/journal.pone.0258594 (PMC8525734; doi:10.1371/journal.pone.0258594)
Supplement: S1 Fig — (PDF) [file pone.0258594.s007.pdf]

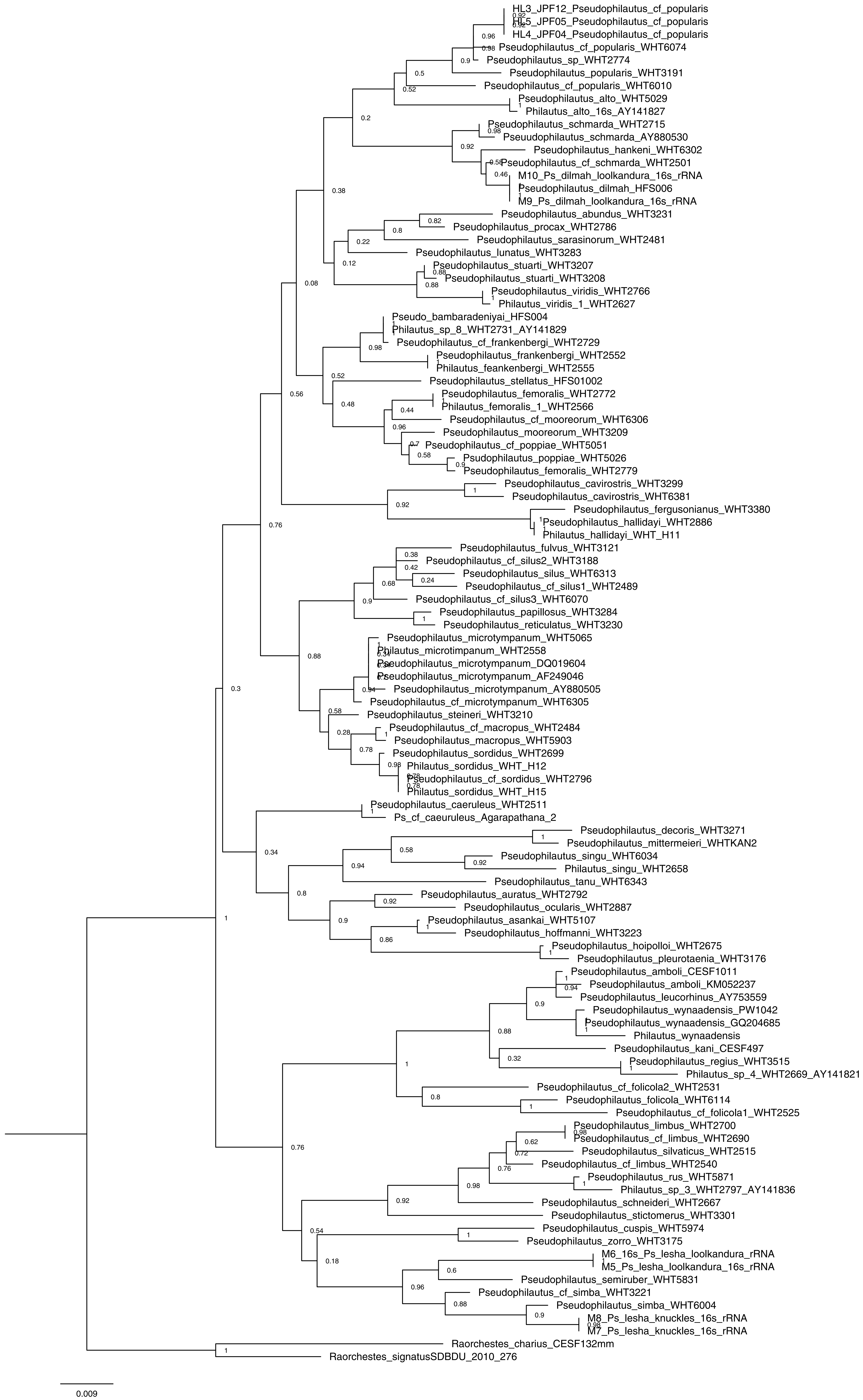

S1 Fig. Molecular phylogenetic relationship of *Pseudophilautus*, based on Maximum likelihood inference of the 16S rRNA + 12S rRNA and Rag1 concatenated data set
