# Supplementary material for: Molecular species delimitation of shrub frogs of the genus Pseudophilautus (Anura, Rhacophoridae)
Source: PLoS One. 2021 Oct 19;16(10):e0258594. doi: 10.1371/journal.pone.0258594 (PMC8525734; doi:10.1371/journal.pone.0258594)
Supplement: S2 Fig — (PDF) [file pone.0258594.s008.pdf]

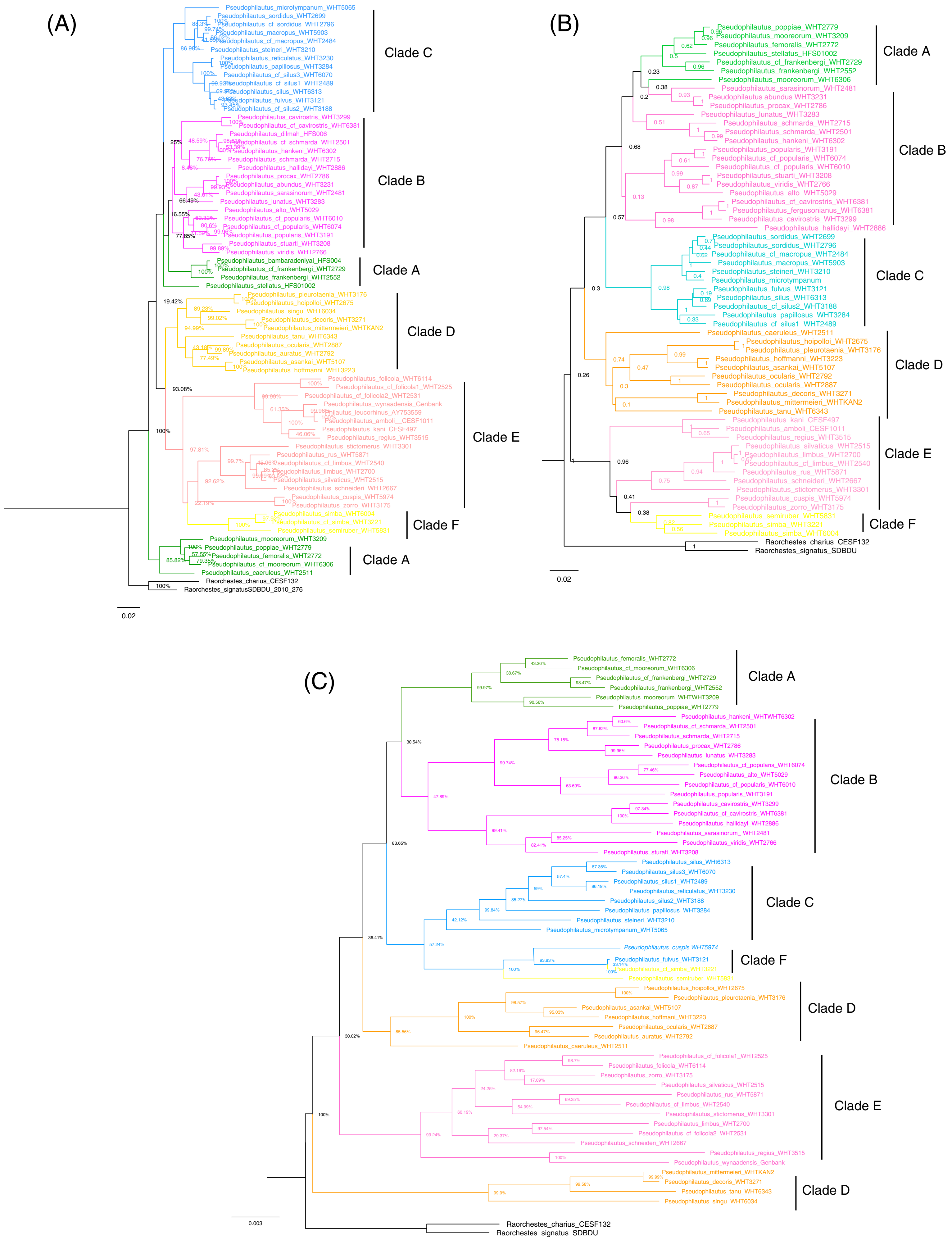

S2 Fig. Molecular phylogenetic relationship of *Pseudophilautus*, based on Bayesian inference of the 16S rRNA (A), 12S rRNA (B) and Rag1 (C) gene fragments.
