# Supplementary material for: Molecular species delimitation of shrub frogs of the genus Pseudophilautus (Anura, Rhacophoridae)
Source: PLoS One. 2021 Oct 19;16(10):e0258594. doi: 10.1371/journal.pone.0258594 (PMC8525734; doi:10.1371/journal.pone.0258594)
Supplement: S3 Fig — (PDF) [file pone.0258594.s009.pdf]

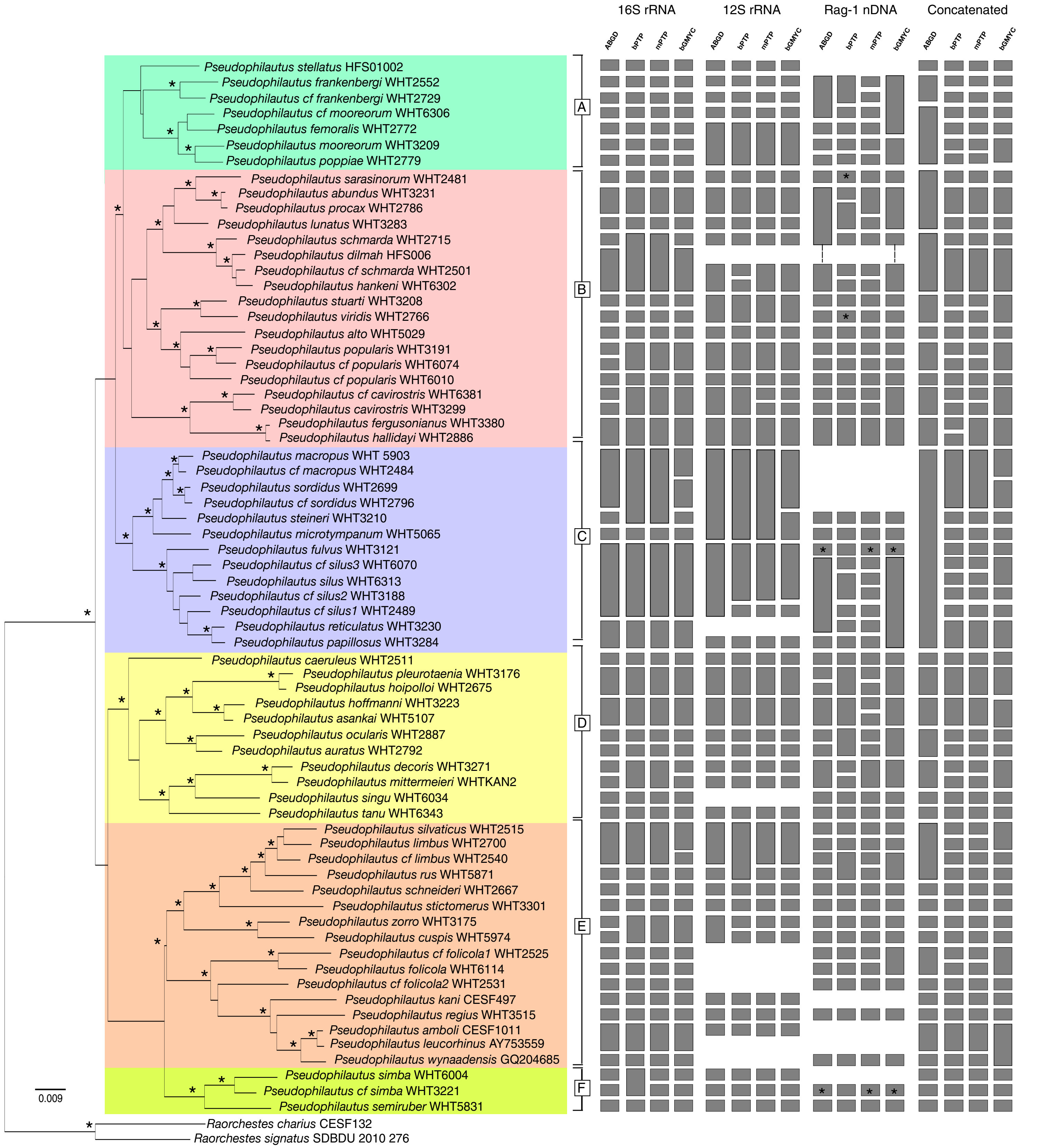

S3 Fig. Recovered species delimitation based on overall significance of results for molecular species delimitation methods (ABGD, bPTP, mPTP and bGMYC) using 16S rRNA, 12S rRNA, Rag-1 nDNA and concatenated data set on only singletons.
